# Telemedicine and cancer care in India: promises, opportunities and caveats

**DOI:** 10.2144/fsoa-2022-0001

**Published:** 2023-01-09

**Authors:** Suryanarayana VS Deo, Raja Pramanik, Meesha Chaturvedi, Anita Nath, Jaya Ghosh, Saroj Kumar Das Majumdar, Naveen Salins, Geeta Kadayaprath, Pankaj K Garg, Arvind Chaturvedi, Sandeep Mathur, Prashant Mathur

**Affiliations:** 1 Department of Surgical Oncology, Dr. B R Ambedkar Institute-Rotary Cancer Hospital, All India Institute of Medical Sciences (AIIMS), Ansari Nagar, New Delhi, 110029, India; 2 ICMR- National Centre for Disease Informatics and Research, Bangalore, Karnataka, 562110, India; 3 Department of Medical Oncology, Tata Memorial Hospital (TMH), Homi Babha Building, Dr Ernest Borges Rd, Parel East, Parel, Mumbai, Maharashtra, 400012, India; 4 Department of Radiotherapy, All India Institute of Medical Sciences (AIIMS), Patra Pada, Bhubaneswar, Odisha 751019, India; 5 Department of Palliative Medicine and Supportive Care, Kasturba Medical College Manipal, Manipal Academy of Higher Education, Manipal, 576104, India; 6 Department of Surgical Oncology, Max Institute of Cancer Care, Max Super Speciality Hospital, 108A, I.P Extension, Patparganj, New Delhi, 110092, India; 7 Department of Surgical Oncology, All India Institute of Medical Sciences (AIIMS), Rishikesh, India; 8 Department of Radiology, Rajiv Gandhi Cancer Institute, Sector – 5, Rohini Institutional Area, Rohini, New Delhi, 110085, India

**Keywords:** Cancer Care continuum, outcomes, survival, technology, tele-oncology

## Abstract

Telemedicine has revolutionized areas of medical practice and care. It has a potential in field of continuum of cancer care in India. SARS-CoV-2 has highlighted the potential use of this tool effectively. Scope of newer applications of telemedicine in field of cancer is reviewed in current paper enlisting benefits to patient, healthcare providers and centers in a developing country like India. Each of them is supported by appropriate evidence and examples. An analysis of strengths and opportunities when compared with weakness and threats brings out how telemedicine can redistribute oncology work force in a rational way and minimize disruption caused by the pandemic. Telemedicine can be utilized in cancer management starting from prevention, screening, diagnosis, treatment and rehabilitation to palliative care.

## Plain language summary

Specialists working for decades in the field of oncology are the best persons to endorse telemedicine, as they can leverage its use to its full potential. The present article is a rigorous review of past literature on telemedicine as well as proposed uses of technologies based on experiences of the authors. It will strengthen promotive, preventive, curative and rehabilitative healthcare delivery.

Telemedicine (TM) uses telecommunications technology as a tool to deliver healthcare to populations with limited access to care [1]. TM has carved a niche for itself in the management of cancer patients in the present pandemic times and it is envisioned to play an ever-increasing role in the coming times also. The present article is a review aimed at improving the judicious integration of TM into current practice of oncology in Indian health set-ups and to bring out the benefits of “telehealth and cancer” for all stakeholders -including doctors, hospitals and patients.

The article also envisages to provide best practices to be followed while providing cancer care and assist in provision of primary healthcare for cancer patients in a country undergoing rapid changes in economy and healthcare

Recent launch of the “Framework for Telemedicine” [2] use in the management of “Cancer, Diabetes, Cardio-vascular diseases and Stroke” by National Centre for Disease Informatics and Research (ICMR), has paved the ground for integration of tele-health technologies spurred by the situation created by COVID -19 pandemic.

## Telemedicine by definition

TM is a means which allows various professionals working in the field of health to undertake evaluation, diagnosis and treatment of patients who are at a distance using various telecommunications technologies. TM has brought through a striking evolution in India and is increasingly becoming a part of healthcare system. Wide spread use of cameras in hand-held mobiles, messenger services and videoconferencing have added a new dimension to TM.

### A brief history of TM applications

The first known use of telehealth took place in the 1940s, when radiology images were sent over telephone lines [3]. Telehealth services gradually expanded in Europe and the USA, but the primary focus was on virtual visits to rural residents to increase their access to medical care. Using telehealth services for years, perhaps even without giving it much thought has been part of practice for all medical practitioners; e.g., getting reports of blood investigations on email/WhatsApp or a doctor accessing test results stored securely in a patient's personal electronic medical record. The scenario really changed since 2020, all over the world, as a response to the COVID-19 pandemic. Many people's perception of telehealth changed during this pandemic when there were nationwide directives to “stay at home” [4]. A new medium of care had to be immediately adopted and accepted to meet the acute and chronic care needs of the patients. In 2020, TM practice guidelines were released by Ministry of Health and Family Welfare (MoHFW) Government of India. The oncology community, both patients and healthcare providers, were quick to integrate it to their plans of care [4].

TM technologies [5] can be broadly classified into two modes ‘synchronous’ and ‘asynchronous’. In the synchronous mode the participants interact with each other dynamically in real time, for example, video consultation. In the asynchronous mode, also called “store and forward” way of communication, the participants can interact or reply in their own time frame. It is mainly used for forwarding the investigation reports or for routine follow-up, for example, email, text-message. Based on the mode of communication, TM can be audio, video or text based. Video consults are better appreciated than audio consults as they improve perception of the genuineness of the therapeutic relationship. Based on the stakeholders or participants of the module, TM can be further classified as patient to healthcare worker (HCW) or one healthcare worker to another.

## Scope of TM in cancer management

Cancer comprises a wide variety of diseases involving different organ systems, each having a specialized multidisciplinary management tailored to his/her performance status. Treatments are often prolonged spanning over several months and need a life-long follow-up. The current Indian healthcare system lacks a structured referral system and therefore cancer patients may present to different types of healthcare setups (spanning from rural primary healthcare centre to a district hospital to a tertiary care hospital).

TM has the potential to improve access and affordability of cancer care in India. It can reduce avoidable hospital visits cutting down the overcrowding in hospitals. It can cut down the barriers of time, space and facilitate multidisciplinary tumor board consultation for patients in remote regions.

TM can be utilized across the cancer care continuum including prevention, screening, diagnosis, treatment, rehabilitation and palliative care. Some of the specific areas are discussed in succeeding paragraphs:

- The first visit “triage”: A patient may contact an oncologist for his complaints and share his investigations and treatment records. Based on these, the oncologist may decide to request further investigations, or fix an appointment for hospital visit with the most appropriate disease management group (DMG). Many patients with advanced cancers and poor performance status may be directly referred to the palliative care department from this screening visit. This tele-triage has a huge potential to cut down the rush at tertiary referral hospitals and at the same time, cutting down the waitlist and streamlining patient management services.
- Tele radiology: Access to Picture Archive and Communication System (PACS) and Digital Imaging and Communications in Medicine (DICOM) images at treating hospitals by remote login access can allow immediate opinions from experts in different institutions. However, conclusive teleradiology opinions may not be available without necessary clinical inputs, Interventional radiology procedures should not be recommended without in person assessment of the clinical condition of the patient.
- Tele-pathology: TM can improve access to advanced onco-pathology services including expert opinions to patients from rural and remote parts of the country using various telepathology technologies. The images can be transmitted in asynchronous mode using “static image-based systems”, “virtual slide systems” or “whole slide imaging (WSI)”. Alternatively, “real-time systems” can be used where the operator remotely guides a robotically controlled motorised microscope.
- The Tumor Board and Treatment Planning: Once the patient has been physically examined, multidisciplinary tumor boards (the standard of care for solid tumors) may be organized on a TM platform to finalize a management plan specific for the case. Hospitals with limited specialists may seek the expert opinion of tumor boards of comprehensive cancer centers (e.g., through the National Cancer Grid (NCG)) [6] using online virtual platforms. Difficult cases may benefit from online molecular tumor boards.
- Preoperative counseling: TM consultations are useful for preoperative optimization of the patients planned for a surgical procedure including pre-operative counselling, discussion of expectations, complications and expenses.
- Monitoring and pre-chemo-cycle assessments: Patients on neoadjuvant and adjuvant chemotherapies who are tolerating their previous cycles well without toxicity may be tele consulted for mid -cycle assessment thus sparing them from the OPD visits to hospitals. Video consults may be done for assessment of common toxicities like mucositis. In patients who have been recently started on a drug and the oncologist is concerned about how well it is being tolerated at home, a video consult may clarify things and ensure adequate tolerance.
- Counseling for radiotherapy: TM can be utilized for counselling and explaining regarding process, planning, expected outcomes, side affects of radiotherapy and for follow-up after completion of radiotherapy.
- Follow-up: Follow-up is an important part of the continuum of cancer care. This is a key area where TM has shown potential.
  a) Patients who have completed their curative treatment and are on regular check-ups typically at a few months' interval with blood investigations and clinical examinations. Such patients may be alternately seen in the clinic and via teleconsultation.
  b) Patients on hormonal therapies who have been stable without toxicities, may be followed using TM and physical consultations alternatively.
  c) Patients on low intensity therapies for some hematological malignancies, tele visits and in person visits may be alternated e.g., a patient of chronic myeloid leukemia in molecular remission on Tyrosine Kinase Inhibitor (TKI e.g., imatinib), low-grade lymphomas on maintenance therapy.
  d) Patients on intensive chemotherapy need intensive close observations for toxicity and supportive care and may not be suitable for teleconsultations. Patients on palliative systemic therapies need physical visits as the disease status continuously evolves over time and the performance status and toxicities vary with time.
  e) In the above scenarios, TM should be used for assessment of symptoms of disease and interpretation of results, prescription of supportive medications allowed in National Medical Council (NMC) guidelines of TN, stoppage of medicines in cases of toxicity and reiteration of advice on diet and exercise. However, a new prescription of anticancer drugs, dose-modifications, and management of oncological medical emergencies, moderate to severe radiation induced reactions should not be attempted on TM.
- Survivorship services: TM can be effectively used for “survivorship services” and “genetic counselling services” and documenting patient reported outcomes (e-PROs) surveys.
- Referral: TM can offer an excellent streamlined solution for appropriate and timely referral of patients to tertiary care cancer centers using standard operating procedures. Following completion of treatment, the same channels and platforms can be used to refer to the primary care physician and follow-up can be ensured. Second opinions from experts can be sought in difficult and challenging situations through TM.
- Oncologic emergencies: Some hospitals in the US are using a modern triage approach for screening patients before they come to the emergency department, e.g., the Jeff Connect at Jefferson Health, Philadelphia. Medical history and symptoms are checked during the video consultation and arrangements are made to meet them at the door when they arrive, escorting them directly into an isolated exam room. Patients also can go straight to the cancer centre, avoiding the emergency department. These can be implemented in the Indian settings, with necessary modifications.
- Wearable technologies, like smartwatches, can be used to monitor vital signs for someone having chemotherapy or radiation therapy for throat cancer to help them decide if intravenous hydration is needed. Tracking calories, steps etc., can help cancer survivors regain control over their deconditioned bodies [7]. Smartphone apps have been developed to support lifestyle changes, as well as to help people take their medications. With the flourishing IT industry in India, custom made apps can be developed which can benefit the implementation of the TM principles for the set up.
- Clinical trials accessibility: Although the pandemic put a temporary halt to some clinical trial enrolments, many studies already underway continued with slight adjustments. Many trial participants are now being monitoring at home using TM and video visits.

### Telemedicine in palliative care

TM in a palliative care setting can be a great source of support to the families caring for patients with terminal illness. An analysis of 15 studies revealed the following findings:

□ It facilitates symptom reporting, assessment and management. It improves management of pain, depression, anxiety, and fatigue and improves psychological distress and improves access to essential symptom control medications [8–12].
□ It can enable multiple family members to participate in a family counselling meeting with the palliative care provider, addressing their anxieties and concerns [13,14]; thereby facilitating planning the end-of-life care [15,16].
□ It fostered communication between patients, families and healthcare providers [17] that enhanced the feelings of safety and security [18].
□ Optimum utilisation of this tele-palliative care can reduce emergency room visits, prevent unnecessary admissions [19,20].
□ Tele-palliative consults foster trust between families and palliative care providers [21], and might relieve the fear and stress of caregivers [22].

## Evidence that telemedicine works in cancer care

A total of nine studies were thoroughly reviewed and the findings were consolidated. With the objective of investigating the usage of smartphone app named (e-PAL) which was based on artificial intelligence (AI), Kamdar *et al.* [23] concluded that pain related to cancer and admission to hospital due to severity of pain can be lowered. Of the 112 people with metastatic cancers in the study, 56 were given the app and the other 56 were assigned to receive usual care. The app would send alerts with daily pain management tips and prompted users to submit their pain rating levels 3 days a week. After 8 weeks, it was reported, that the severity of pain had reduced by 20% in persons using the app and the chances of admission to hospital due to pain had reduced by 69% in persons who used the app.

Another study showed that using a web-based symptom reporting system (STAR) [24] that alerts the care team about problems leads to actions that alleviate suffering and improve patient outcomes. Study participants included patients with cancers of breast, lung, gynaecologic cancers and genitourinary cancers in advanced stages. These patients were receiving chemotherapy on an outpatient basis. Participants were divided into two groups; one which used symptom reporting system (STAR); and another group which discussed symptoms with oncologists during their usual visits. It was observed that the group using the web-based system had lived longer by 5 months as a median (31.2 months) when compared with those not using the tool (26 months). The twelve commonest symptoms were dyspnoea, tiredness, hot flashes, pain, nausea and loss of appetite. These symptoms were graded in five-point scale by the STAR group on a weekly basis.

Dolittle *et al.* [25], in 1997, compared the operational costs of three different types of oncology practice: a TM clinic and a fly-in outreach clinic, both held in rural areas, and a traditional clinic held in a city hospital, and found tele-oncology to be cost effective. Thaker *et al.* [26] conducted a cost analysis of a TM model for cancer care (tele-oncology) in northern Queensland, Australia, compared with the usual model of care. They reported that the tele-oncology model of care resulted in net savings, mainly due to avoidance of travel costs. Such savings could be redirected to enhancing rural resources and service capabilities. In fact, some studies have reported higher patient satisfaction with virtual visits. Weinstein *et al.* [27] demonstrated the efficacy of bundling of teleradiology, telepathology, and tele-oncology in breast cancer care. Medical literature is replete with evidence of comparable satisfaction and efficacy of telephonic genetic counselling as compared with in-person genetic counselling. This provides a strong rationale for the effectiveness of tele-cancer genetics services. Bradbury *et al.* [28] showed that the “Remote Videoconferencing Tele-Genetic Delivery Model” successfully identifies genetic carriers and yields high levels of patient satisfaction. Jhaveri *et al.* [29] showed that under the “Queensland Remote Chemotherapy Supervision model (QReCS)” selected chemotherapy regimens could be administered in rural hospitals by rural based generalist doctors and nurses, under the supervision of medical oncologists and chemotherapy competent nurses through videoconferencing. These models rely on physical examination by local primary healthcare staff reiterating the importance of HCW-HCW TM. The mHealth technologies [30], often linked to smart phone apps, broadly include texting and messaging efforts that provide patients with ongoing engagement, support and coaching.

## Barriers & facilitators of implementation of telemedicine in India: an analysis

A systematic analysis of strengths, weaknesses, opportunities and threats was carried out for implementation of TM in a vast country like India (Table 1). Strengths and opportunities far outweigh the weaknesses and threats in the Indian scenario.

**Table 1.** SWOT analysis of use of telemedicine in India.

**Strengths** 1. India is a populous country where TM in the field of cancer could enhance reach of the healthcare professional to grass root level, i.e., rural pockets, which do not have easy access to tertiary level healthcare 2. TM can enhance virtual availability of oncologists of various streams, i.e., medical, surgical and histopathologists to a larger set of population in a developing country like India (where doctor patient ratios are lower than many countries) 3. Advanced cancer is a multisystemic disease which can give rise to emergency medical situations. Accelerated communications with the help of TM has the potential to save lives with the right prescription of the treating doctor within time 4. Wide application of TM can play a major role in prioritizing patient care	**Weakness** 1. There is an advancement in technologies every day, so TM usage needs to be updated with advancement of technology. This may be a challenge in India due to capital expenditure involved and recurring costs 2. Training and capacity building for correct usage of TM in India would be a challenge especially for a disease like cancer which needs specialised training 3. Connectivity with high speed internet at all times is a pre-requisite which is still a challenge faced in Indian rural set-ups 4. A tele medical consultation in the presence of an unknown technician who is facilitating the consultation may cause apprehensions regarding the authenticity of treatment offered/advised, especially in a conservative society with several languages and dialects 5. Sub-site-specific guidelines would be required for leading sites of cancer in India to enable diagnosis of correct anatomical site of cancer, e.g., head and neck cancers
**Opportunities** 1. Most of the cancers in India are diagnosed at locally advanced stage. TM provides a huge opportunity for early diagnosis, improve the quality of life and chances of survival of such advanced stage cancers 2. TMis the most economically viable option with wide usage of mobile devices & internet connectivity. Factors which can bring down cost are – travel costs by the patient and relatives to hospitals, infrastructure costs of hospitals i.e. disposables, bed linen usage, personal protection equipment (PPE), waiting spaces 3. Widespread use of TM gives an opportunity to downstage cancer in India. Earlier stages at detection and early initiation of treatment gives an opportunity to potentially cure cancer 4. In rural parts of the country, effectiveness of health system response maybe improved by linking TM with other modes of care delivery (e.g., supplies of medicines, involving Accredited Social Health Activists -ASHA)	**Threats** 1. There are situations where lack of clinical examination may miss out several important findings for a disease like cancer, i.e., pleural effusion which maybe elicited clinically can be missed out in a case of cancer lung 2. The linguistic disparities and firm socio-cultural beliefs followed in India may hinder wider implementation of telemedicine 3. Ethical issues may arise where prescription of certain analgesics is involved for advance stages of cancer through tele medical consultation 4. Patient consent needs to be ensured, electronic data protected and credentials of healthcare provider need to be verified for all teleconsultations

## Limitations & practical challenges: cancer telemedicine

Clinicians need to appreciate that TM is not well suited for discussing sensitive information [2]. Any discussion of prognosis, or treatment plans needs the physical presence and proximity of the clinician. If used inappropriately, there is a possibility that patients and their next of kin get the impression that their clinicians are insensitive and uncaring. From the perspective of healthcare providers, the challenges involved with providing telehealth include choosing and learning to use a platform, making sure there is proper information technology support and figuring out how to deal with scheduling glitches. Training with the TM technology is essential to facilitate rapport, maximize engagement and conduct an accurate virtual exam. Thus, training oncologist and their support staff, in the usage of modern telehealth technologies is highly desirable. Stable internet connectivity is an actual issue in many remote regions in India. Cancer patients of lower socio-economic status face a bigger challenge to get connected through internet. Other important concerns include -those requiring physical presence at clinics (e.g., oncology emergency) may resort to virtual consultation while other patients may get convinced only after making physical visit to oncologist despite having minor ailment.

Early signs of relapse or recurrence could be detected by routine clinical examinations which is not appropriate if carried out from a distance. Subsequent investigations to detect any potential signs may get delayed.

The prime concern is selection of patients who need to be physically present at the clinic and those who can have a follow -up at home.

Finally, the security perception due to physical interaction with physician which acta as a catalyst in motivating the patient is reduced during use of tele-medicine.

## Integration into present system in India

TM technology can serve to redistribute the oncology work force in a rational way, where needed and minimize the disruption caused by the disease. It can be a catalyst to organize our cancer care delivery system.

Development of a user-friendly interface ‘e-Sanjeevani’ [31] facilitated interaction of medical professionals and patients in rural and urban environments. This was a browser-based application which found its optimum use in recent times. Though, use of ‘e-Sanjeevani’ in the field of oncology has to be guarded, indirect benefits through departments of radiology and pathology can be useful at arriving at a diagnosis.

Widespread availability of 4G/3G internet networks, affordable data packages, widespread use of smart phones by people of all sections of the Indian society reiterate the potential of use of these tele-oncology technologies in the field of cancer prevention, diagnosis, and management in India. The Prime Minister Aatmanirbhar Swasth Bharat Yojana aims at developing the capacities of the primary, secondary, and tertiary health sector. The scheme envisages to expand the Integrated Health Information Portal to all the union territories and states to connect all public health labs, which will be an important step in the integration of TM in the present healthcare system in India.

## Conclusion

Cancer is rapidly emerging as a significant non-communicable diseases in low- and middle-income countries (LMICs). There are challenges of optimal cancer care delivery due to shortage of human resource and cancer care facilities in India. During last decade and especially during COVID pandemic, India has witnessed a surge in the use of TM technologies.

## Future perspective

Policy mandates have supported this activity unanimously. It is time to realise its full potential, integrate into clinical practice, make it more patient as well as doctor friendly. It is predicted that TM, in the next decade, would bring back the ‘house call’ of the old, shifting the focal point of care from hospitals and offices to the place where he/she resides This will pave the way ahead for Universal Health Coverage.

**Executive summary**

- Indian healthcare system lacks a structured referral system and therefore cancer patients may present to different types of healthcare setups (spanning from rural primary healthcare center to a district hospital to a corporate tertiary care hospital).
- Use of TM in oncology across the cancer care continuum ranges from prevention, screening, diagnosis, treatment, and rehabilitation to palliative care.
  ○ The first visit ‘Triage’: Based on these, the oncologist may decide to request further investigations, or fix an appointment for hospital visit with the most appropriate disease management group (DMG). Tele triaged patients with advanced cancers and poor performance status may directly go to the palliative care.
  ○ Tumor board and treatment planning: As a next step, multidisciplinary tumor boards can opine to finalize a management plan. Difficult cases may benefit from online molecular tumor boards.
  ○ Teleradiology: Access to Picture Archive and Communication System (PACS) and Digital Imaging and Communications in Medicine (DICOM)images at treating hospitals by remote login access can allow immediate opinions from experts in different institutions.
  ○ Telepathology: Images can be transmitted in asynchronous mode using ‘static image-based systems’, ‘virtual slide systems’ or ‘whole slide imaging (WSI)’. Else ‘real-time systems’ can be used where the operator remotely guides a robotically controlled motorised microscope.
  ○ Preoperative counseling: These are useful for preoperative optimization of the patients planned for a surgical procedure including pre-operative counseling, discussion of expectations, complications and expenses.
  ○ Monitoring and pre-chemo-cycle assessments: Patients on neoadjuvant and adjuvant chemotherapies who are tolerating their previous cycles well without toxicity may be tele consulted for mid -cycle assessment thus sparing them from the OPD visits. Video consults may be done for assessment of common toxicities like mucositis.
  ○ Counseling for radiotherapy: Utilization for counselling on process, planning, expected outcomes, side affects of radiotherapy and for follow-up after completion of radiotherapy.
  ○ Survivorship services: Effectively used for ‘genetic counselling services’ and documenting patient reported outcomes (e-PROs) surveys.
  ○ Referral: Following completion of treatment, the same channels and platforms can be used to refer to the primary care physician. Second opinions from experts can be sought in difficult and challenging situations.
  ○ Palliative care: Tele-palliative consults foster trust between families and palliative care providers and might relieve the fear and stress of caregivers.
  ○ Wearable technologies, like smartwatches, can be used to monitor vital signs for someone having chemotherapy or radiation therapy for throat cancer and this the data from can help them decide if intravenous hydration is needed.
